# Supplementary material for: Rapid prototyping and design of cybergenetic single-cell controllers
Source: Nat Commun. 2021 Sep 24;12:5651. doi: 10.1038/s41467-021-25754-6 (PMC8463601; doi:10.1038/s41467-021-25754-6)
Supplement: Supplementary file 1 — Supplementary Information [file 41467_2021_25754_MOESM1_ESM.pdf]

Supplementary Information
Rapid Prototyping and Design of Cybergenetic Single-Cell Controllers

Sant Kumar, Mark Rullan, Mustafa Khammash

S1 Theoretical analysis

S1.1 Autocatalytic Integral Controller

The reactions involved in this biomolecular controller reaction network are:

$$\begin{aligned} V &\xrightarrow{\alpha\mu} V{+}V \\ V + X_L &\xrightarrow{\alpha} X_L \\ V &\xrightarrow{k} V + X_1 \end{aligned}$$

The dynamics of V can be given by the following ODE (Ordinary Differential Equation):

$$\dot{V} = \alpha\mu V - \alpha V X_L. \tag{S1}$$

At steady state,

$$\begin{aligned} \dot{V} &= 0 \\ \Rightarrow \alpha\mu V - \alpha V X_L &= 0 \\ \Rightarrow \alpha V(\mu - X_L) &= 0 \\ \Rightarrow V = 0 \text{ (absorbing state) } \text{ or } \; X_L &= \mu. \end{aligned} \tag{S2}$$

Therefore, this system reaches steady-state either when X_L (the controlled species) is equal to μ (the setpoint) or when V is zero. As shown in [1], $V = 0$ equilibrium point is always unstable while the other $X_L = \mu$ equillibrium point is locally asymptotically stable under certain conditions on parameter α.

In a stochastic setting, the dynamics of the first moment of the controller species V can be described by:

$$\dot{\mathbb{E}[V]} = \alpha\mu\mathbb{E}[V] - \alpha\mathbb{E}[VX_L]. \tag{S3}$$

We can simplify the equation above by using:

$$\mathbb{E}[VX_L] = \mathbb{E}[V]\mathbb{E}[X_L] + Cov(X_L, V), \tag{S4}$$

where $Cov(X_L, V)$ is the covariance between X_L and V. The time evolution of $\mathbb{E}[V]$ is then reduced to the following expression:

$$\begin{aligned} \dot{\mathbb{E}[V]} &= \alpha\mu\mathbb{E}[V] - \alpha(\mathbb{E}[V]\mathbb{E}[X_L] + Cov(X_L, V)) \\ &= \alpha\mathbb{E}[V]\left(\mu - \mathbb{E}[X_L] - \frac{Cov(X_L, V)}{\mathbb{E}[V]}\right). \end{aligned} \tag{S5}$$

At steady state,

$$\begin{aligned} \dot{\mathbb{E}[V]} &= 0 \\ \Rightarrow \alpha\mathbb{E}[V]\left(\mu - \mathbb{E}[X_L] - \frac{Cov(X_L, V)}{\mathbb{E}[V]}\right) &= 0 \\ \Rightarrow \mathbb{E}[V] = 0 \text{ or } \mathbb{E}[X_L] &= \mu - \frac{Cov(X_L, V)}{\mathbb{E}[V]}. \end{aligned} \tag{S6}$$

This indicates that even if $\mathbb{E}[V]$ is non-zero, the expected value of X_L at steady-state will settle to the set-point μ only when the covariance between X_L and V is zero. But one can approximate the steady-state value $\mathbb{E}[X_L]$ to the set-point μ when the copy-number of V at steady-state is large enough (given bounded $Cov(X_L, V)$). In the limit $\mathbb{E}[V] \to \infty$, the system converges to the deterministic solution (Supplementary Equation S2), which shows perfect tracking [1].

S1.1.1 Basal production of controller species

$$\phi \xrightarrow{\gamma} V$$

The introduction of a basal production term γ modifies the dynamics in the following manner:

$$\dot{\mathbb{E}[V]} = \alpha\mu\mathbb{E}[V] - \alpha\mathbb{E}[VX_L] + \gamma. \tag{S7}$$

Using Supplementary Equation 4, this reduces to the following expression:

$$\begin{aligned} \dot{\mathbb{E}[V]} &= \alpha\mu\mathbb{E}[V] - \alpha(\mathbb{E}[V]\mathbb{E}[X_L] + Cov(X_L, V)) + \gamma \\ &= \alpha\mathbb{E}[V]\left(\mu - \mathbb{E}[X_L] - \frac{Cov(X_L, V)}{\mathbb{E}[V]} + \frac{\gamma}{\alpha\mathbb{E}[V]}\right). \end{aligned} \tag{S8}$$

Again, at steady state,

$$\begin{aligned} \dot{\mathbb{E}[V]} &= 0 \\ \Rightarrow \alpha\mathbb{E}[V]\left(\mu - \mathbb{E}[X_L] - \frac{Cov(X_L, V)}{\mathbb{E}[V]} + \frac{\gamma}{\alpha\mathbb{E}[V]}\right) &= 0 \\ \Rightarrow \mathbb{E}[V] = 0 \ \text{ or } \ \mathbb{E}[X_L] &= \mu - \frac{Cov(X_L, V)}{\mathbb{E}[V]} + \frac{\gamma}{\alpha\mathbb{E}[V]}, \end{aligned} \tag{S9}$$

$$\text{Offset} \ = \ -\frac{Cov(X_L, V)}{\mathbb{E}[V]} + \frac{\gamma}{\alpha\mathbb{E}[V]}. \tag{S10}$$

As observed in Figure 2 (in the main text), the addition of a basal production reaction for controller species V eliminates the absorbing state of the system under stochastic setting. This does induce an offset in the output at steady-state (Supplementary Equation S9). This offset is composed of two separate terms $-\frac{Cov(X_L,V)}{\mathbb{E}[V]}$ and $\frac{\gamma}{\alpha\mathbb{E}[V]}$. Through further analysis of our experimental data (Supplementary Figure S1), we observed that the basal production rate (γ) affects both terms as decreasing γ resulted in a decrease in the value of both terms, thus in turn led to a decrease in the offset.

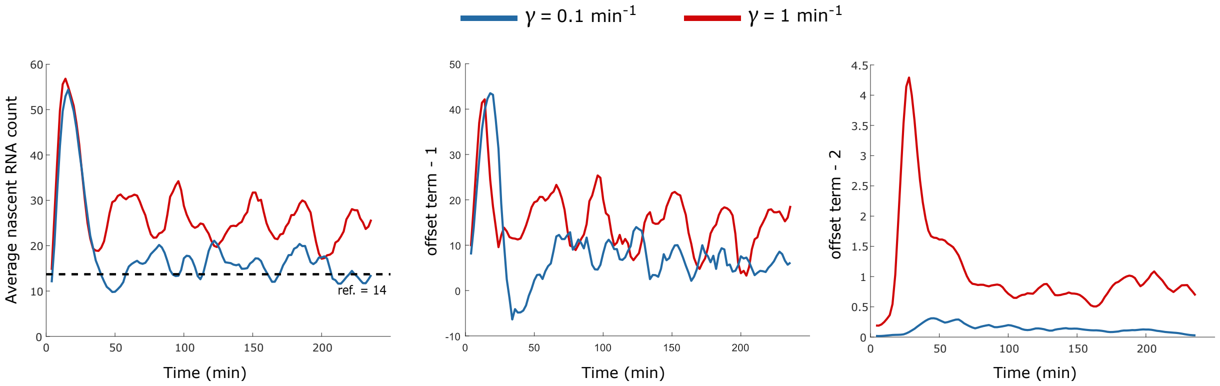

Supplementary Figure S1: Effect of basal production (leakiness) controller species V on the Autocatalytic Integral Controller performance - Decrease in basal production rate leads to a decrease in the steady-state error/offset in the output. Left: time-course evolution of average nascent RNA counts for the controller with two different basal production rates. Center: time-course evolution of the first offset-term, $-\frac{Cov(X_L,V)}{\mathbb{E}[V]}$. Right: time-course evolution of the second offset-term, $\frac{\gamma}{\alpha\mathbb{E}[V]}$. Top: steady-state error (absolute value) distribution of nascent RNA counts for all the cells. (Experimental parameters: $k = 0.005\,\text{min}^{-1}, \alpha = 0.01\,\text{min}^{-1}$, initial $V = 300$ and $\mu = 14$). Source data are provided as a Source Data file.

S1.2 Antithetic Integral Controller

The reactions involved in this controller motif are:

$$\phi \xrightarrow{\mu} Z_1$$
$$\phi \xrightarrow{\theta X_L} Z_2$$
$$\phi \xrightarrow{k Z_1} X_1$$
$$Z_1 + Z_2 \xrightarrow{\eta} \phi$$

The first order moment dynamics of the controller species Z_1 and Z_2 can be described by:

$$\mathbb{E}[\dot{Z_1}] = \mu - \eta \mathbb{E}[Z_1 Z_2] \tag{S11}$$
$$\mathbb{E}[\dot{Z_2}] = \theta \mathbb{E}[X_L] - \eta \mathbb{E}[Z_1 Z_2]. \tag{S12}$$

At steady state,

$$\begin{aligned} \mathbb{E}[\dot{Z_1}] = 0, \ \ \mathbb{E}[\dot{Z_2}] = 0 \\ \Rightarrow \mu - \eta \mathbb{E}[Z_1 Z_2] = \theta \mathbb{E}[X_L] - \eta \mathbb{E}[Z_1 Z_2] = 0 \\ \Rightarrow \mathbb{E}[X_L] = \frac{\mu}{\theta}. \end{aligned} \tag{S13}$$

This implies that, as explained in [2], the expected value of X_L will robustly track the set-point $\frac{\mu}{\theta}$. This result is obtained with the assumption that there is no degradation of controller species Z_1 and Z_2.

S1.2.1 Dilution of controller species

$$Z_1 \xrightarrow{\delta} \phi$$
$$Z_2 \xrightarrow{\delta} \phi$$

In fast dividing cells, dilution leads to a notable degradation of controller species. This violates the assumption needed for the above mentioned result (Supplementary Equation S13). After including the dilution effect, the first order moment dynamics of the controller species can be re-written as follows:

$$\mathbb{E}[\dot{Z_1}] = \mu - \eta \mathbb{E}[Z_1 Z_2] - \delta \mathbb{E}[Z_1] \tag{S14}$$
$$\mathbb{E}[\dot{Z_2}] = \theta \mathbb{E}[X_L] - \eta \mathbb{E}[Z_1 Z_2] - \delta \mathbb{E}[Z_2], \tag{S15}$$

where λ is the dilution rate corresponding to the doubling time of the host cell. Again, at steady state,

$$\begin{aligned} \mathbb{E}[\dot{Z_1}] = 0, \ \ \mathbb{E}[\dot{Z_2}] = 0 \\ \Rightarrow \mu - \eta \mathbb{E}[Z_1 Z_2] - \delta \mathbb{E}[Z_1] = \theta \mathbb{E}[X_L] - \eta \mathbb{E}[Z_1 Z_2] - \delta \mathbb{E}[Z_2] = 0 \\ \Rightarrow \frac{\mu}{\theta} - \mathbb{E}[X_L] - \frac{\delta}{\theta}\left(\mathbb{E}[Z_1] - \mathbb{E}[Z_2]\right) = 0 \\ \Rightarrow \mathbb{E}[X_L] = \frac{\mu}{\theta} - \underbrace{\frac{\delta}{\theta}\left(\mathbb{E}[Z_1] - \mathbb{E}[Z_2]\right)}_{\text{Offset}}. \end{aligned} \tag{S16}$$

From the above equation we can easily deduce that the dilution rate δ generates a deviation from the set-point $\frac{\mu}{\theta}$. This offset is proportional to the difference in copy-number between Z_1 and Z_2, as well as inversely proportional to the value of θ. Furthermore, the offset also depends on the dilution rate itself. In our experiments (Supplementary Figure S2), we observed that increasing the dilution/degradation rate (δ) of the controller species Z_1 and Z_2 led to an increase in the deviation from the set-point for a given set of parameters.

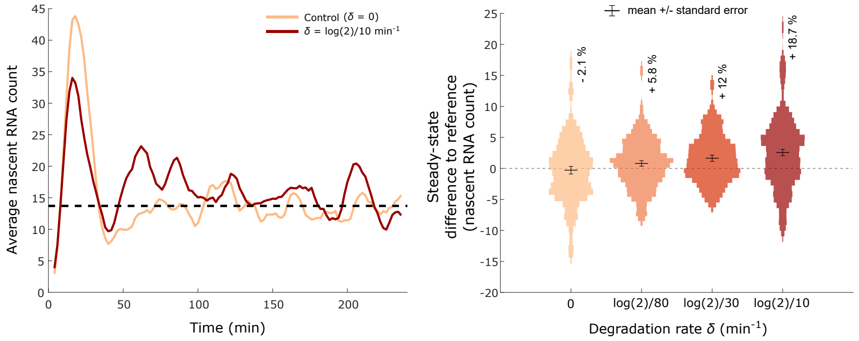

Supplementary Figure S2: Effect of degradation of controller species Z_1 and Z_2 on the Antithetic Integral Controller performance - Increase in degradation rate leads to a marginal increase in the steady-state error/offset in the output. Left: time-course evolution of average nascent RNA counts for the controller with and without degradation. Right: contains the distribution of steady-state errors for four experiments with different degradation rates. Inset percentage values are the percentage mean errors from the reference. Degradation rate of $log(2)/80$ min^{-1} corresponds to 80 min doubling time of the intended host cell-type which is *Saccharomyces cerevisiae* in our experiments. Mean +/- standard error is indicated for all four distributions. (Experimental parameters: $k = 0.1$ min^{-1}, $\eta = 5$ min^{-1}, $\theta = 0.02$ min^{-1} and $\mu = 14 \times \theta$ min^{-1}). Source data are provided as a Source Data file.

S2 Demonstration with different target system

One key feature of the proposed Cyberloop framework is that it can be easily and quickly adapted to work with different cellular systems or different biological target networks to be controlled. To demonstrate this we considered the engineered strain of *Saccharomyces cerevisiae* presented in [3]. This strain has a blue light-inducible VP-EL222 expression system and a nuclear translocation reporter dPSTR system. Together they provide the required optogenetic control and measurement of the gene expression in *Saccharomyces cerevisiae*. Here, blue light induces the localization of RFP in the nucleus increasing nuclear fluorescence relative to the fluorescence in the cytoplasm (the reader is referred to Figure 3 in [3] for further details). This is totally different from the target system used in our study where nascent RNA count was the controlled output.

We employed the Antithetic Integral control motif to control the fluorescence localized in the nucleus (relative to the whole cell fluorescence). The output from a single cell is given by:

$$\text{Single cell output} \quad = \quad \frac{\text{Mean nuclear fluorescence}}{\text{Mean fluorescence of the whole cell}}$$

As expected, this control motif was able to bring the average output across cells to the desired set-points (Supplementary Figure S3).

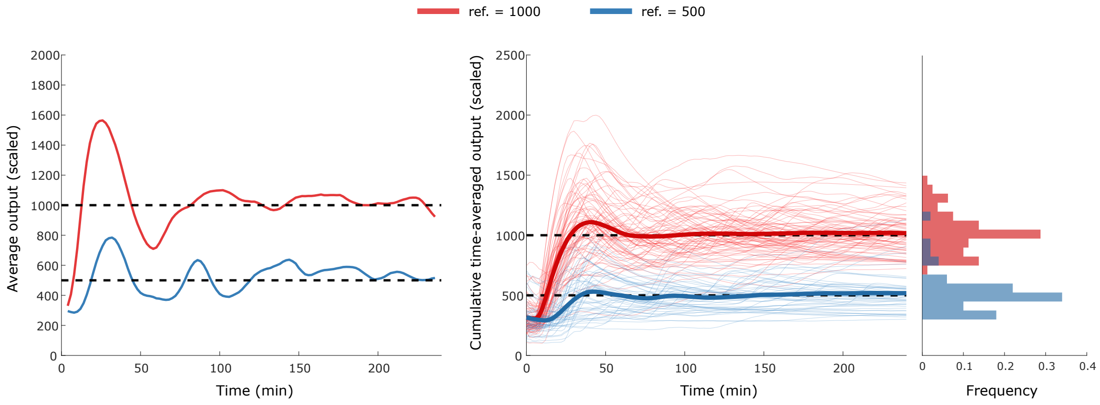

Supplementary Figure S3: Set-point tracking with Antithetic Integral control motif in a nuclear localization output system. Left: time-course evolution of average output of all the cells in two different set-point experiments. Center: thin lines represent the evolution of cumulative time averages of the output in individual cells, and thick lines are the population average. Right: distribution of the cellular output over the course of the two experiments. Source data are provided as a Source Data file.

S3 Additional figures

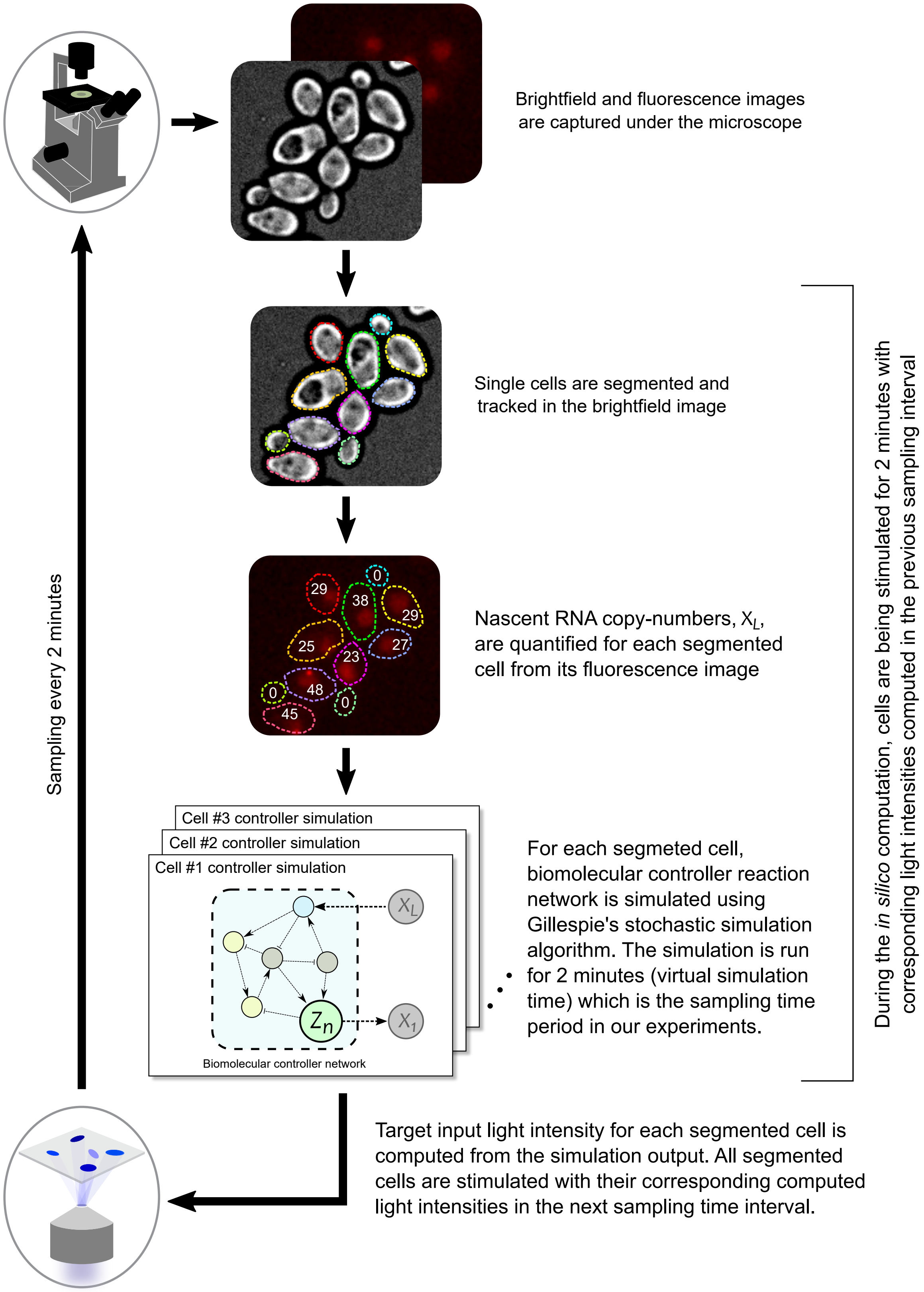

Supplementary Figure S4: Detailed *in silico* steps in the Cyberloop framework.

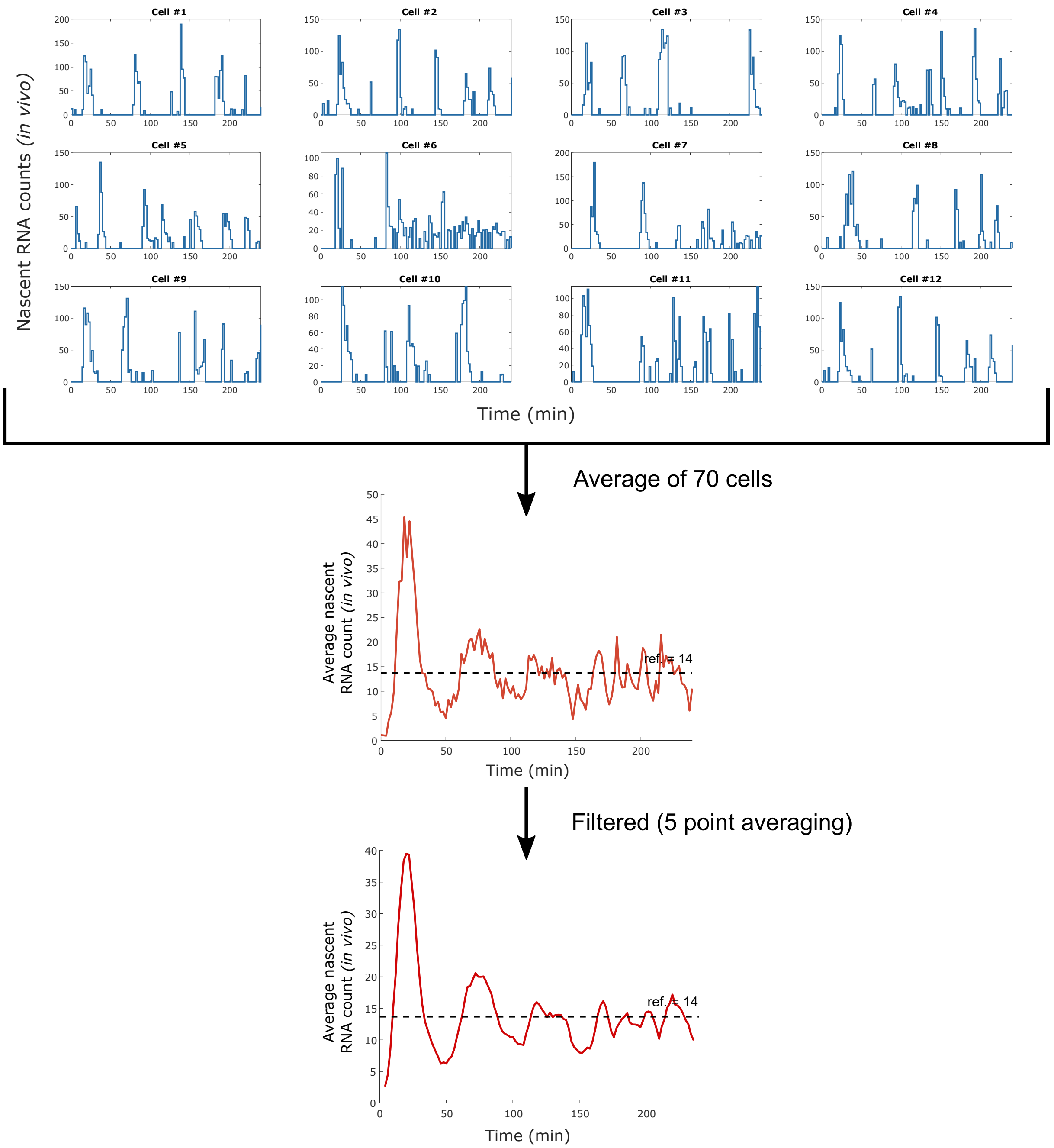

Supplementary Figure S5: Single cell traces and averaged data from a Cyberloop experiment (Figure 5a, red, main text). Top: time-course evolution of nascent RNA counts of 16 individual cells in the experiment. As transcription occurs in bursts, nascent RNAs are also observed as showing bursting behaviour and highly stochastic dynamics [4]. Center: Average of single-cell nascent RNA count traces of 70 cells being tracked/targeted throughout the experiment. Bottom: applying 5 point averaging filter to the mean trajectory. We have only reported this filtered trajectory in all the results presented in the main text. Source data are provided as a Source Data file.

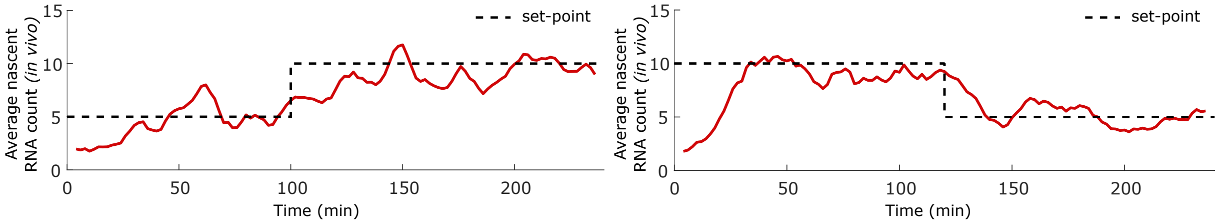

Supplementary Figure S6: Demonstration of dynamic set-point tracking with Antithetic Integral Control motif (Figure 3a, main text). Plots show the time-course evolution of average nascent RNA counts in two separate Cyberloop experiments. Left: set-point is increased from 5 to 10 at 100 minutes during the experiment. Right: set-point is decreased from 10 to 5 at 120 minutes during the experiment (Experimental parameters: $k = 0.015\ \text{min}^{-1}$, $\eta = 5\ \text{min}^{-1}$, $\theta = 0.02\ \text{min}^{-1}$ and $\mu = 5 \times \theta$ (for set-point 5) or $10 \times \theta$ (for set-point 10) min^{-1}; Number of cells: left - 84, right - 88). Source data are provided as a Source Data file.

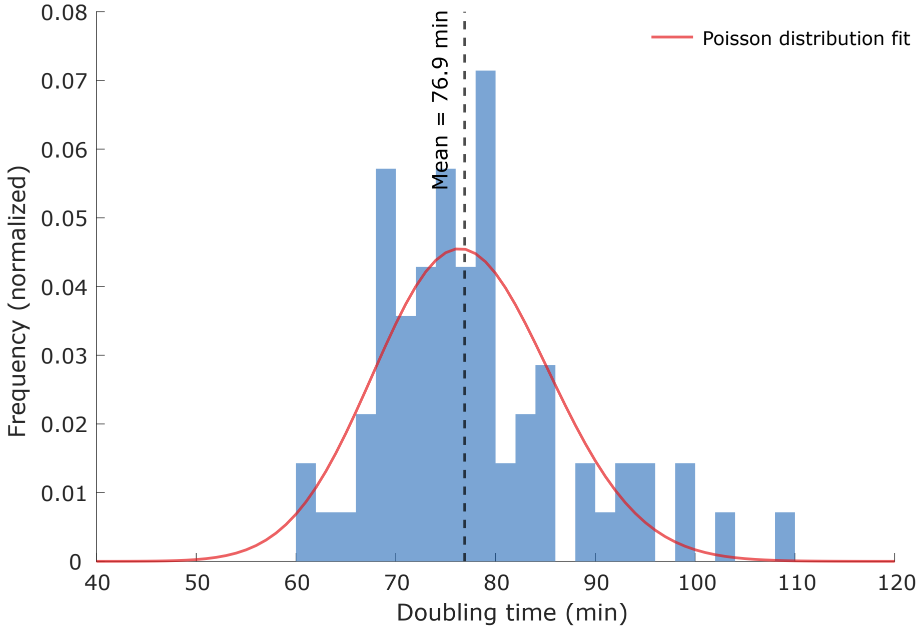

Supplementary Figure S7: Distribution of doubling times of 70 cells. Cells were manually picked from different experiments, and their doubling times were calculated by manually observing them through time-lapse images. For the results presented in Figure 4c in the main text, doubling times for the dilution rate needed for single-cell *in silico* controllers were sampled from the poisson distribution fit shown here as the red curve. Source data are provided as a Source Data file.

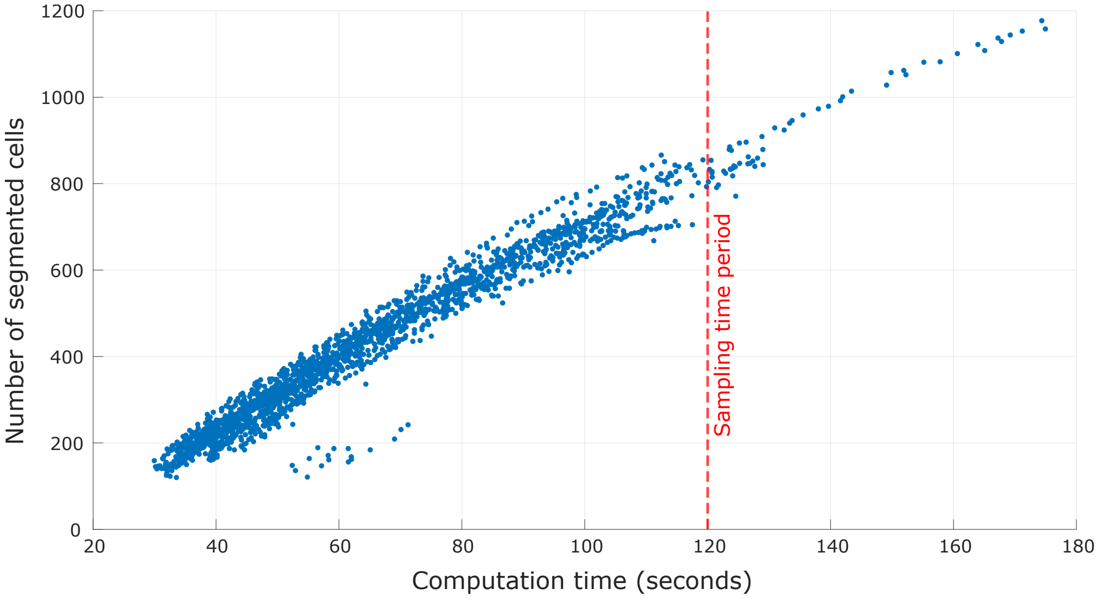

Supplementary Figure S8: For all the Cyberloop experiments presented in this study, we have used a sampling time period of 2 minutes. The limiting factor in deciding this time period in our experiments has been the computation time needed to run cell segmentation, cell tracking, quantification and stochastic controller simulation routines. In these experiments, we have used the software tools developed in [4] (based on [5] and [6]) for segmentation, tracking and quantification of individual cells. Our software framework is sequential and thus the computation time is directly proportional to the number of cells segmented in the microscopy image, as seen in this figure (showing 2057 data points). If needed, a shorter sampling time can be achieved by using parallel processing for segmentation, tracking, and quantification. Source data are provided as a Source Data file.

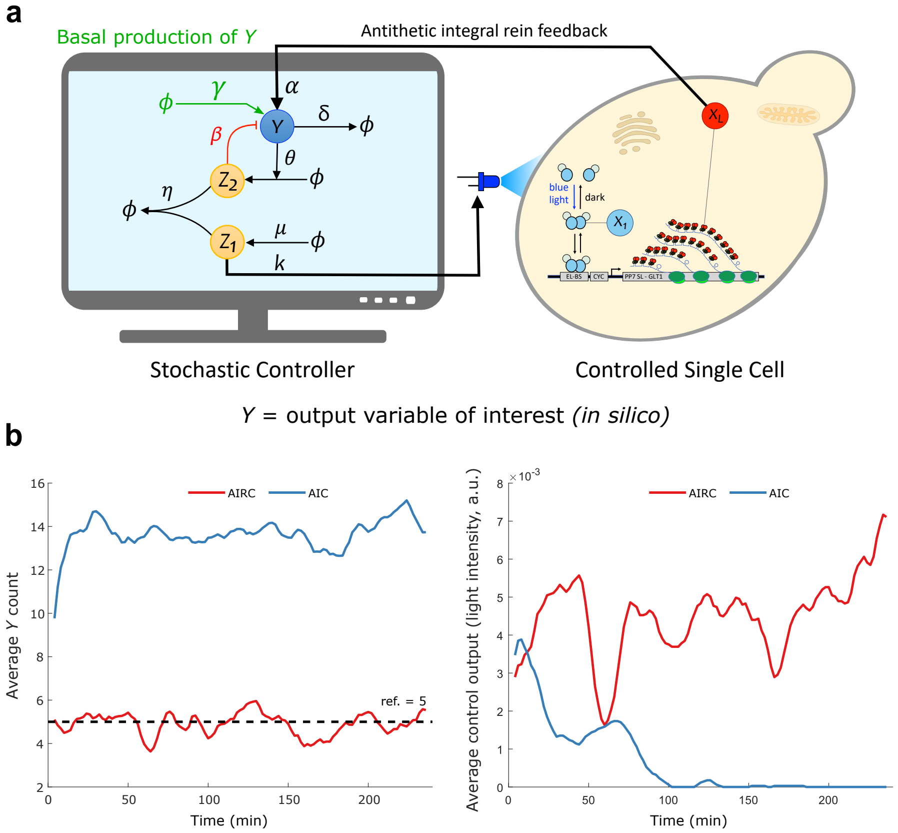

Supplementary Figure S9: Antithetic Integral Rein Controller (AIRC) in Cyberloop. **(a)** Addition of a basal production reaction (shown in green color) of the output molecule Y (added to the setup shown in Figure 4b in the main text). **(b)** Two Cyberloop experiments, one with Antithetic Integral Control (AIC) motif ($\beta = 0$) and another with AIRC motif ($\beta \neq 0$) having same values for the other parameters, were carried out. Left: shows the time-course evolution of average output Y abundance. Right: shows the time-course evolution of average control-output (light intensity) during the course of these two experiments (Experimental parameters: $k = 0.01\,\text{min}^{-1}$, $\eta = 5\,\text{min}^{-1}$, $\theta = 0.02\,\text{min}^{-1}$ and $\mu = 5 \times \theta\,\text{min}^{-1}$, $\alpha = 0.5\,\text{min}^{-1}$, $\delta = 0.2\,\text{min}^{-1}$, $\gamma = 2\,\text{min}^{-1}$ and $\beta = (0, 5)\,\text{min}^{-1}$; Number of cells: red - 65, blue - 68). Under these parameters, the AIRC motif is able to bring and keep the output molecule Y to the desired reference level while the AIC motif is unable to achieve set-point tracking. This is due to the presence of a high basal production rate of the output molecule Y. This additional basal production reaction in the controlled system network violates the operating condition (Theorem 2 in [2]) required for AIC motif to control the given network. Addition of a rein feedback (AIRC) to the Antithetic motif restores the robust perfect adaptation capability of the controller under such conditions. Source data are provided as a Source Data file.

References

[1] Corentin Briat, Christoph Zechner, and Mustafa Khammash. Design of a synthetic integral feedback circuit: Dynamic analysis and dna implementation. *ACS Synthetic Biology*, 5(10):1108–1116, 2016.

[2] Corentin Briat, Ankit Gupta, and Mustafa Khammash. Antithetic integral feedback ensures robust perfect adaptation in noisy biomolecular networks. *Cell Systems*, 2(2):133, 2016.

[3] Melinda L. Perkins, Dirk Benzinger, Murat Arcak, and Mustafa Khammash. Cell-in-the-loop pattern formation with optogenetically emulated cell-to-cell signaling. *Nature Communications*, 11:1355, 2020.

[4] Marc Rullan, Dirk Benzinger, Gregor W. Schmidt, Andreas Milias-Argeitis, and Mustafa Khammash. An optogenetic platform for real-time, single-cell interrogation of stochastic transcriptional regulation. *Molecular Cell*, 70(4):745–756, 2018.

[5] S. Dimopoulos, C. E. Mayer, F. Rudolf, and J. Stelling. Accurate cell segmentation in microscopy images using membrane patterns. *Bioinformatics*, 30(18):2644–2651, 2014.

[6] M. Ricicova, M. Hamidi, A. Quiring, A. Niemistö, E. Emberly, and C. L. Hansen. Dissecting genealogy and cell cycle as sources of cell-to-cell variability in mapk signaling using high-throughput lineage tracking. *Proceedings of the National Academy of Sciences of the United States of America*, 110(28): 11403–8, 2013.
