# Supplementary material for: Rapid prototyping and design of cybergenetic single-cell controllers
Source: Nat Commun. 2021 Sep 24;12:5651. doi: 10.1038/s41467-021-25754-6 (PMC8463601; doi:10.1038/s41467-021-25754-6)
Supplement: Supplementary file 2 — Reporting Summary [file 41467_2021_25754_MOESM2_ESM.pdf]

nature research

Corresponding author(s): Professor Mustafa Khammash

Last updated by author(s): Aug 23, 2021

Reporting Summary

Nature Research wishes to improve the reproducibility of the work that we publish. This form provides structure for consistency and transparency in reporting. For further information on Nature Research policies, see our Editorial Policies and the Editorial Policy Checklist.

Statistics

For all statistical analyses, confirm that the following items are present in the figure legend, table legend, main text, or Methods section.

n/a	Confirmed	
☐	☒	The exact sample size (n) for each experimental group/condition, given as a discrete number and unit of measurement
☐	☒	A statement on whether measurements were taken from distinct samples or whether the same sample was measured repeatedly
☒	☐	The statistical test(s) used AND whether they are one- or two-sided<br>*Only common tests should be described solely by name; describe more complex techniques in the Methods section.*
☐	☒	A description of all covariates tested
☒	☐	A description of any assumptions or corrections, such as tests of normality and adjustment for multiple comparisons
☐	☒	A full description of the statistical parameters including central tendency (e.g. means) or other basic estimates (e.g. regression coefficient) AND variation (e.g. standard deviation) or associated estimates of uncertainty (e.g. confidence intervals)
☒	☐	For null hypothesis testing, the test statistic (e.g. F, t, r) with confidence intervals, effect sizes, degrees of freedom and P value noted<br>*Give P values as exact values whenever suitable.*
☒	☐	For Bayesian analysis, information on the choice of priors and Markov chain Monte Carlo settings
☒	☐	For hierarchical and complex designs, identification of the appropriate level for tests and full reporting of outcomes
☒	☐	Estimates of effect sizes (e.g. Cohen's d, Pearson's r), indicating how they were calculated

Our web collection on statistics for biologists contains articles on many of the points above.

Software and code

Policy information about availability of computer code

Data collection	1. YouScope 2.0 (build: R2016-01, open source) -- Used for microscope and projector control with configuration files developed by Rullan and Benzinger et al. (2018).<br>2. MATLAB R2016a (academic use) -- Used for cell segmentation, cell tracking, fluorescence quantification, and stochastic simulation (Gillespie's Stochastic Simulation Algorithm) of biomolecular controllers. Segmentation, tracking and quantification were carried out using software tools developed by Rullan and Benzinger et al. (2018) which are based on Dimopoulos et al. (2014) and Ricicova et al. (2013).
Data analysis	All analyses in this study were performed on MATLAB R2018a (academic use) platform using custom scripts. Manuscript figures were structured and formatted on Inkscape (v0.92, open source).

For manuscripts utilizing custom algorithms or software that are central to the research but not yet described in published literature, software must be made available to editors and reviewers. We strongly encourage code deposition in a community repository (e.g. GitHub). See the Nature Research guidelines for submitting code & software for further information.

Data

Policy information about availability of data

All manuscripts must include a data availability statement. This statement should provide the following information, where applicable:

- Accession codes, unique identifiers, or web links for publicly available datasets
- A list of figures that have associated raw data
- A description of any restrictions on data availability

Raw data for all the results/figures presented in this article, including Supplementary figures, are available in the Source Data file. Any additional data are available upon request.

Field-specific reporting

Please select the one below that is the best fit for your research. If you are not sure, read the appropriate sections before making your selection.

- [x] Life sciences
- [ ] Behavioural & social sciences
- [ ] Ecological, evolutionary & environmental sciences

For a reference copy of the document with all sections, see nature.com/documents/nr-reporting-summary-flat.pdf

Life sciences study design

All studies must disclose on these points even when the disclosure is negative.

Sample size	For the experiments in this study, sample size refers to the number of cells tracked/stimulated for the full duration of the experiment. Approximately 84 cells on an average were targeted and tracked in each experiment. The upper limit on number of tracked cells was set to be 140. This was chosen to allow a sampling time of 2 minutes. Tracking more cells would result in more time taken for computation between sampling, and thus will require a larger sampling time period (Supplementary Figure S8). Please refer to Rullan and Benzinger et al. (2018) for more details.
Data exclusions	There were few instances in which the agarose pad started drifting during the experiment, resulting in a drift of cells in the field of view. And, there were few experiments where the perfect focus system of the microscope was unable to maintain the same focus for the full duration of the experiment. We have excluded data from those experiments in our final analysis.
Replication	In the Cyberloop setup, each cell is observed, controlled and targeted independently (segmented, tracked and quantified separately). Each cell has its own independent in silico controller which computes the input light intensity for the cell, and the DMD projection hardware targets only that particular cell with the computed blue light intensity. On an average 84 independent cells were targeted and tracked separately in each experiment. Experiments in Figure 5a and Figure 5e (right) were performed twice on separate days, and similar results were obtained from both sets of experiments.
Randomization	All cells within a particular region in the field of view (under the microscope) were considered for the experiment. This region was chosen based on cell density on the sample plane. As mentioned before, upper limit on number of tracked cells was set to be 140. This target region was chosen so that there were roughly that many cells inside the region. Cells were allowed to settle for 1 hour (without any stimulation) before starting the experiment.
Blinding	Researchers were not blinded during the study because the experiment routine, data processing and analysis were automated. Experiments were performed with cells randomly sampled from culture with no selection bias.

Reporting for specific materials, systems and methods

We require information from authors about some types of materials, experimental systems and methods used in many studies. Here, indicate whether each material, system or method listed is relevant to your study. If you are not sure if a list item applies to your research, read the appropriate section before selecting a response.

Materials & experimental systems

n/a	Involved in the study	
x		Antibodies
x		Eukaryotic cell lines
x		Palaeontology and archaeology
x		Animals and other organisms
x		Human research participants
x		Clinical data
x		Dual use research of concern

Methods

n/a	Involved in the study	
x		ChIP-seq
x		Flow cytometry
x		MRI-based neuroimaging
